# Water-soluble contrast agents in adhesional small bowel obstruction: meta-analysis and PRECIS-2 assessment of trials

**DOI:** 10.1093/bjsopen/zraf049

**Published:** 2025-05-09

**Authors:** Matthew Gowell, Daniel M Baker, Greta McLachlan, David N Naumann, Adam Peckham-Cooper, Neil J Smart, Matthew J Lee

**Affiliations:** Department of Trauma and Emergency General Surgery, University Hospitals Birmingham NHS Foundation Trust, Queen Elizabeth Hospital Birmingham, Birmingham, UK; Leeds Institute for Emegency Surgery, Leeds Teaching Hospitals NHS Trust, Leeds, UK; Department of Surgery, Frimley Health Foundation Trust, Frimley Park Hospital, Camberley, UK; Department of Trauma and Emergency General Surgery, University Hospitals Birmingham NHS Foundation Trust, Queen Elizabeth Hospital Birmingham, Birmingham, UK; Department of Inflammation and Ageing, University of Birmingham, Birmingham, UK; Academic Department of Military Surgery and Trauma, Royal Centre for Defence Medicine, Queen Elizabeth Hospital Birmingham, Birmingham, UK; Leeds Institute for Emegency Surgery, Leeds Teaching Hospitals NHS Trust, Leeds, UK; Department of Colorectal Surgery, Royal Devon and Exeter NHS Foundation Trust, Exeter, UK; Department of Trauma and Emergency General Surgery, University Hospitals Birmingham NHS Foundation Trust, Queen Elizabeth Hospital Birmingham, Birmingham, UK; Department of Applied Health Sciences, College of Medicine and Health, University of Birmingham, Birmingham, UK

## Abstract

**Background:**

Adhesional small bowel obstruction is a common presentation to acute general surgical services. Initial management is typically conservative and includes the use of water-soluble contrast agents. Current trials assessing water-soluble contrast agents are limited by sample size and demonstrate contrasting results. The aim of this review was to systematically appraise the use of water-soluble contrast agents in adhesional small bowel obstruction.

**Methods:**

This systematic review and meta-analysis was registered with PROSPERO (CRD42024573136) and conducted in line with PRISMA guidelines. Searches of Medline, Embase and Central databases were undertaken to include randomized clinical trials reporting the use of water-soluble contrast agents in adhesional small bowel obstruction. Searches were last updated on 26 July 2024. The primary outcome was the need for operative intervention. Secondary outcomes included the rate of intestinal ischaemia, the need for bowel resection, and mortality. A random-effects meta-analysis was conducted for outcomes reported in three or more studies. Risk of bias was assessed using the Cochrane Risk-of-Bias tool, and trial methods were appraised using the PRagmatic Explanatory Continuum Indicator Summary (PRECIS-2) tool.

**Results:**

In all, 11 randomized controlled trials were included with a median sample size of 88 (range 26–242), nine of which were single-centre studies; only one study used computed tomography imaging to diagnoses adhesional small bowel obstruction. Meta-analysis revealed no significant difference in operative intervention (odds ratio 0.63, 95% confidence interval 0.39 to 1.01; *P* = 0.053), small bowel ischaemia, small bowel resection, or mortality. Risk of bias raised concerns in several domains. PRECIS-2 assessment showed trials were pragmatic rather than explanatory designs.

**Conclusion:**

This review does not support the use of therapeutic water-soluble contrast agents in adhesional small bowel obstruction. Further adequately powered trials are needed. Standardization of diagnostic modality and consideration of explanatory designs should be considered.

## Teaser

An updated meta-analysis showed that water-soluble contrast agents are not therapeutic for adhesive small bowel obstruction. A review of trials showed statistical fragility. PRECIS assessment revealed pragmatic, rather than explanatory, trials.

## Introduction

Following major abdominal surgery, there is approximately a one in ten chance that a patient develops adhesional small bowel obstruction (aSBO)^1^. Given the high global volume of major abdominal surgery, it is not surprising that aSBO is a major cause of intestinal obstruction, accounting for approximately 50% of all cases^2^. This is reflected by the high cost and burden felt in health systems. In the USA, aSBO accounts for around 300 000 admissions and costs of US$1.3 billion each year^3^. In the absence of evidence of ischaemia, treatment is typically supportive, because it is recognized that surgical treatment of aSBO may lead to the development of more adhesions, creating a cycle of surgical intervention^4^.

One of the adjuncts to non-operative management of aSBO is Gastrografin (Bayer AG, Leverkusen, Germany), a water-soluble contrast agent (WSCA). Gastrografin is highly osmotic (approximately 1900 mOsmol/L)^5^ and supports treatment in two ways. First, it is thought that osmotic effects draw fluid from the bowel wall into the lumen, relieving obstruction^5^. Second, serial radiological imaging can identify contrast beyond the small bowel (for example, in the colon and rectum), indicating likely success of conservative measures^6^. The use of Gastrografin was first described in trials in the early 1990s^7^ and has been recommended as first-line treatment for aSBO^4^. Recent global shortages in Gastrografin^8^ have highlighted the need for a reappraisal of evidence.

The aims of the present study were to systematically appraise the literature regarding the use of Gastrografin and related compounds in aSBO, reassessing clinical outcomes, and to review methodological aspects of trial design.

## Methods

### Study design

This systematic review and meta-analysis was conducted with reference to the Cochrane Handbook^9^. It was prospectively registered on PROSPERO (CRD42024573136) and reported according to PRISMA guidance^10^.

### PICO and eligibility criteria

The population of interest was adult patients undergoing initial non-operative management of aSBO. Populations with partial or resolving aSBO at the time of entry were not eligible. The interventions of interest were amidotrizoate/diatriazote iodine containing WSCA, typically marketed as Gastrografin or Urografin, given as part of initial non-operative management. For the purpose of this review, these interventions are referred to as WSCA. The interventions could be given at any time following diagnosis. Acceptable comparators were ‘standard’ non-operative management with or without placebo. The primary outcome of interest was the rate of surgical intervention.

Randomized trials reporting the use of oral/enteral WSCA in adult patients diagnosed with aSBO were eligible for inclusion. Studies were required to compare WSCA against standard clinical care (observation with nasogastric drainage) or placebo in order to be included. Studies not published in English were excluded because no translation resource was available.

### Information sources

The MEDLINE, Embase, and Central databases were searched using a search strategy developed with an information specialist. The search strategy included key search terms relating to disease, study design, and the intervention under study (for example, intestinal obstruction, Gastrografin, randomized trial). A sample search strategy is presented in *Appendix S1*. Searches were last updated on 26 July 2024.

### Selection process

Search results were uploaded to Covidence (Veritas Health Innovation, Melbourne, Victoria, Australia). Each abstract was independently screened by two authors (combination of M.G., D.M.B., and G.M.), with conflicts resolved by the senior author (M.J.L.). Full texts were retrieved and again independently assessed by two authors. Conflicts were resolved by discussion with a third (senior) author.

### Data collection process

Data from included studies were extracted into a data extraction sheet on the Covidence platform. This was completed independently by two reviewers (combination of two of M.G., D.M.B., and G.M.) and compared before completion of data capture.

### Data items

Data collected included first author and year of publication. Key demographics and mode of diagnosis of aSBO were captured. Information regarding intervention characteristics (dosing and timing) and gastric drainage was collated.

### Outcomes

Clinical outcomes of interest were the need for surgery following treatment with WSCA, the occurrence of bowel resection and intestinal ischaemia in those undergoing surgery, and inpatient mortality.

### Study risk of bias assessments

Risk of bias was assessed using the Cochrane risk-of-bias tool. This was completed by two authors as previously stated and reviewed by a third senior author (A.P.-C./D.N.N./M.J.L.).

### Data analysis

A random effects meta-analysis was performed using the Mantel–Haenszel technique for each outcome of interest where three or more studies reported the outcomes. This calculated risk difference for each outcome, and was presented with subgroups according to how small bowel obstruction (SBO) was diagnosed. The summary statistic was presented as an odds ratio (OR) with 95% confidence interval (95% c.i.). In addition, fragility and reverse fragility was calculated for the key outcome of need for surgery. Analyses were conducted using R version 4.4.1 (www.r-project.org). Specifically, the R packages ‘meta’^11^ and ‘fragility’^12^ were used.

### Reporting bias assessments

An additional assessment of trial external validity was undertaken using the PRagmatic Explanatory Continuum Indicator Summary (PRECIS-2) tool^13^. This tool assesses trial conduct across nine domains, rating each from 1 (entirely explanatory design) to 5 (entirely pragmatic design). Studies were independently rated using the PRECIS-2 too by three reviewers (A.P.-C., D.N.N., and M.J.L.), with a median score used to summarise results. An additional value reporting the median across all domains was also calculated, giving an overall appraisal of the study.

### Influence of bias and sensitivity analysis

The influence of publication bias was undertaken using a visual assessment of a funnel plot generated using Egger’s test for the primary outcome of interest of the review (occurrence of surgery). Outlying studies were excluded, and the random effects model was reassessed. Explorations of aspects of bias were also conducted. Where a Cochrane bias domain had three or more studies with a low risk of bias reported, the meta-analysis was rerun excluding studies at unclear, medium, or high risk for that domain.

## Results

### Study selection

**Fig. 1:**
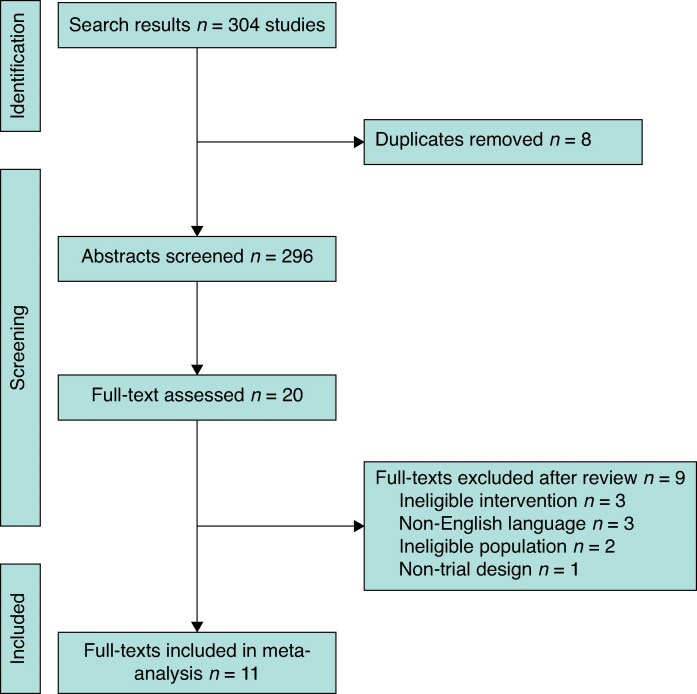
PRISMA flow chart

Initial searches identified 304 potentially eligible studies. Following the removal of duplicates, 296 studies were screened and 20 full texts were assessed for eligibility. During the review of full texts, three studies were excluded for an ineligible intervention, three for non-English language publications, two for ineligible populations, and one for non-trial design. Finally, 11 randomized trials were included in the systematic review (*Fig. 1*). A summary of exclusion reasons for key studies is presented in *Table S1*.

### Study characteristics

Of the 11 included studies, nine were single-centre trials^14–22^, and two were multicentre trials^23,24^. There was a median sample size of 88 (range 26–242). One study used computed tomography diagnosis (CT) of aSBO as an eligibility criterion^24^, whereas the remaining studies used abdominal X-ray (AXR). All but two studies compared WSCA to standard care. The studies of Burge *et al.*^17^ and Khorshidi *et al.*^21^ used a placebo of 0.9% saline administered via the nasogastric tube. Where specified, the timing of receipt of intervention varied from immediately after randomization^15,18^ to 4 days after admission^22^. There was variable reporting on the management of nasogastric tubes around the intervention. Of the five studies reporting nasogastric tube management, two reported on drainage of the stomach prior to administration^16,24^. The time of nasogastric tube clamping following drug administration ranged from 1 h^17,20^ to 3 h^16,18^. Summary study and patient characteristics are presented in *Table 1*.

**Table 1:** Study characteristics

Author (year)	Country	Setting	Funding	Sample size	Participant age* in years		Mode of diagnosis	Intervention and dose	Comparator	Stated primary outcome
					Intervention	Control				
Farid (2010)^14^	Egypt	Single centre	No funding	110	45(14.6)	45.8(15.5)	AXR	Gastrografin 100 ml, NG clamped for 2 h	Standard care	Resolution of symptoms or operative management
Haule (2013)^15^	Uganda	Single centre	Wellcome Trust	50	N/A	N/A	AXR	Gastrografin 100 ml after randomization	Standard care	Number requiring surgery
Rahmani (2013)^22^	Iran	Single centre	No funding	84	53.17(18.23)	50.14(15.36)	AXR	Gastrografin 100 ml within 4 days after admission	Standard care	Clinical or radiological improvement
Biondo (2003)^16^	Spain	Single centre	No funding	90	60(15.5)	65.6(14.2)	AXR	Gastrografin 100 ml immediately after diagnosis	Standard care	Number requiring non-operative *versus* surgical treatment
								NG suctioned prior to administration and clamped for 3 h		
Burge (2005)^17^	New Zealand	Single centre	No funding	37	70 (34–97)	73 (41–99)	AXR	Gastrografin 100 ml, NG clamped for 1 h	Placebo (isotonic saline) 100 ml	Time to resolution of aSBO (flatus and bowel motion)
Kumar (2009)^18^	India	Single centre	No funding	41	40.48(14.96)	43.4(16.33)	AXR	Gastrografin 60 ml after randomization, NG clamped for 2–3 h	Standard care	Number reaching clinical relief of SBO
Fevang (2000)^19^	Norway	Single centre	No funding	98	N/A	N/A	AXR	Mixture of Gastrografin 100 ml and barium 100 ml	Standard care	Resolution of symptoms or operative management
Scotté (2017)^24^	France	Multicentre	Picardie Regional Council	242	62 (51–77)	65 (51–79)	CT	Gastrografin 100 ml via NG	Standard care	Need for operative intervention within 48 h of randomization
								NG drained 2 h before and clamped for 2 h after		
Lee (2004)^20^	Hong Kong	Single centre	No funding	150	64 (16–90)	66 (17–87)	AXR	Urografin 50–100 ml after randomization, NG clamped for 1 h	Standard care	Need for operative intervention
Di Saverio (2008)^23^	Italy	Multicentre	No funding	76	63.7 (18.5)	67.7 (15.2)	AXR	Gastrografin 150 ml diluted with 50 ml water	Standard care	Need for operative intervention
Khorshidi (2019)^21^	Turkey	Single centre	No funding	26	56.6(10.7)	58.6(12.2)	AXR	Gastrografin 100 ml, NG clamped for 2 h	Placebo (0.9% saline) 100 ml	Resolution at 48 h

*Participant age is given as the mean(standard deviation) or median (interquartile range). N/A, not available; AXR, abdominal X-ray; CT, computed tomography; NG, nasogastric tube; aSBO, adhesional small bowel obstruction; SBO, small bowel obstruction.

### Risk of bias

Risk of bias assessment yielded significant concerns across several domains (*Table 2*). Allocation concealment was not undertaken in any study that compared WSCA to standard care, because there was no placebo intervention, and repeated abdominal radiography was performed (therefore blinding was not possible). It is not clear who undertook outcome assessments in the majority of cases. There was inconsistency in outcome reporting, which may reflect selective reporting. Time frames for ‘time to surgery’ varied from within 48 h to any time during the hospital stay. Other causes of bias were frequently judged as being high risk. This was due, in part, to frequent statements of ‘resident/surgeon discretion to operate’, adding a subjective element to factors influencing the primary outcome of interest.

**Table 2:** Risk of bias

Author (year)	Sequence generation	Allocation concealment	Blinding of participants and personnel	Blinding of outcome assessors	Incomplete outcome data	Selective outcome reporting	Other sources of bias
Farid (2010)^14^	Unclear	High	High	Unclear	Unclear	Unclear	High
Haule (2013)^15^	Low	High	High	Unclear	Unclear	Unclear	High
Rahmani (2013)^22^	Unclear	Unclear	High	High	High	High	High
Biondo (2003)^16^	High	High	High	High	High	Unclear	Unclear
Burge (2005)^17^	Low	Low	Low	Unclear	Unclear	Unclear	High
Kumar (2009)^18^	High	High	High	High	Low	High	No judgement
Fevang (2000)^19^	Unclear	High	High	High	Unclear	Unclear	High
Scotté (2017)^24^	Low	Low	High	Unclear	Low	Low	Unclear
Lee (2004)^20^	Low	Unclear	Unclear	Unclear	Low	Low	Unclear
Di Saverio (2008)^23^	Low	Low	Unclear	Unclear	Low	High	High
Khorshidi (2019)^21^	Unclear	Unclear	Unclear	Unclear	High	High	Unclear

### Data synthesis

**Fig. 2:**
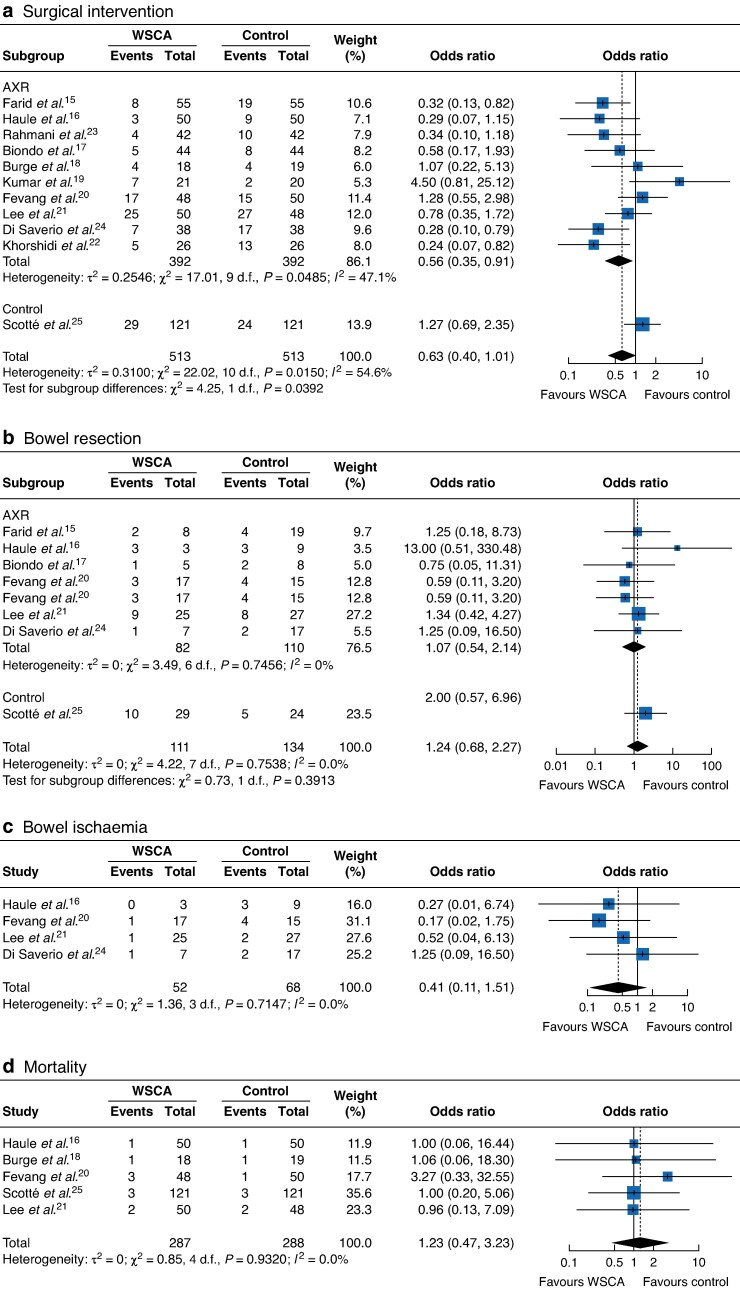
Results of meta-analyses **a** Rates of surgical intervention. **b** Rates of bowel resection. **c** Rates of bowel ischaemia. **d** Mortality. A Mantel–Haenszel random-effects model was used for meta-analysis. Odds ratios are shown with 95% confidence intervals. WSCA, water-soluble contrast agent; AXR, abdominal X-ray.

Meta-analysis of the impact of WSCA on rates of surgical intervention showed no significant reduction in the rates of surgical intervention after WSCA in 11 studies (OR 0.63, 95% c.i. 0.39 to 1.01; *P* = 0.053). Within the AXR subgroup, only one study (Kumar *et al.*^18^) showed an increased rate of intervention after WSCA. In the trial using CT diagnosis^24^, the OR of intervention was 1.27 (95% c.i. 0.69 to 2.34), which was different to the pooled rates of intervention in the AXR group (*P* = 0.039). There was no significant difference in rates of resection after WSCA across eight studies (OR 1.24, 95% c.i. 0.68 to 2.27; *P* = 0.484). Similarly, there was no significant difference between groups in the rate of small bowel ischaemia at surgery across five studies (OR 0.41, 95% c.i. 0.11 to 1.51; *P* = 0.181). Finally, there was no difference in mortality rates (OR 1.27, 95% c.i. 0.47 to 3.23; *P* = 0.676). Forest plots are shown in *Fig. 2*.

### Fragility and reverse fragility

**Fig. 3:**
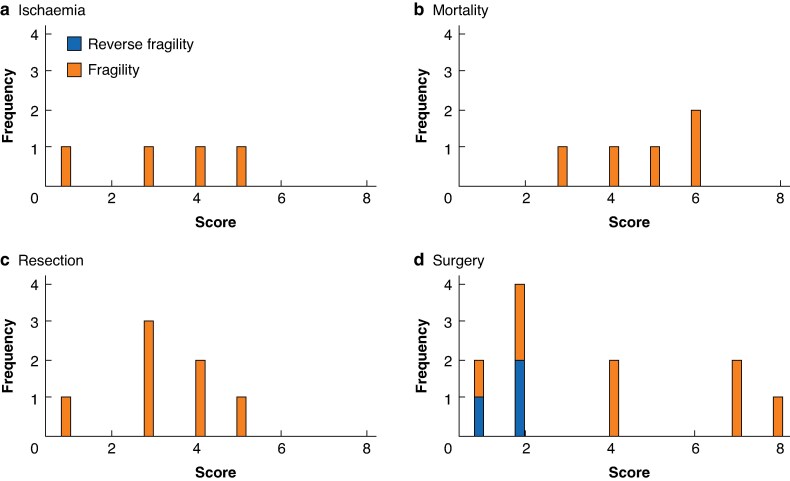
Fragility and reverse fragility plots for outcomes **a** Ischaemia. **b** Mortality. **c** Resection. **d** Surgery. Frequency refers to number of studies with that fragility/reverse fragility index.

Fragility assessment on the need for surgery showed a median fragility index of 4 (interquartile range (i.q.r.) 2–7). When fragility assessment was repeated for the need for bowel resection, the median fragility index was 3 (i.q.r. 1–5). For four studies reporting ischaemia, the fragility index ranged from 1–5, and the median fragility index for mortality 4 (range 3–6). Plots of fragility and reverse fragility for outcomes are shown in *Fig. 3*.

### Applicability

**Fig. 4:**
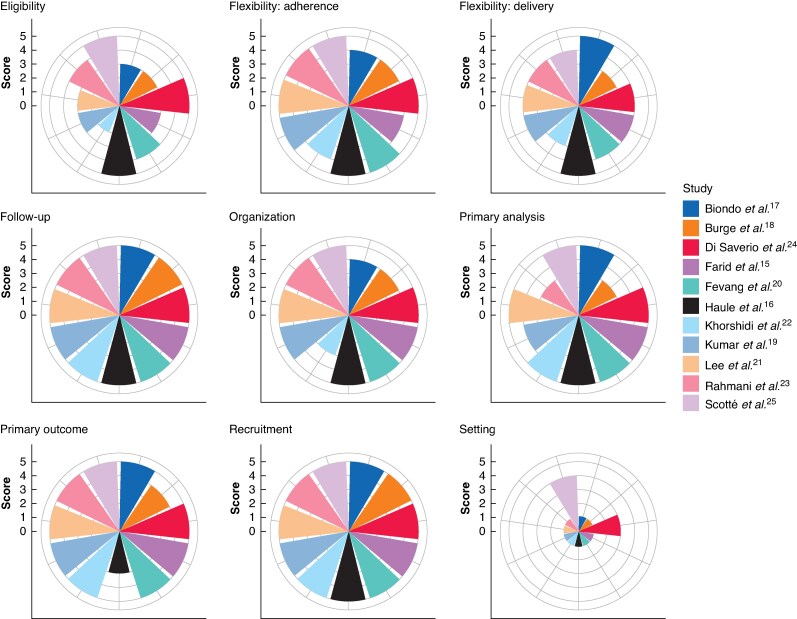
PRagmatic Explanatory Continuum Indicator Summary (PRECIS) wheels demonstrating rating of each study according to PRECIS domain

When the PRECIS-2 tool was used to assess applicability, there was a tendency towards pragmatic designs of trials, with median scores ≥4 in all domains except for setting, which had a median score of 1 (reflecting the predominance of single-centre designs). All studies had a median score of 5 across domains, except for the study of Burge *et al*.^17^, which had a median score of 4. PRECIS wheels for each study are shown in *Fig. 4*.

### Sensitivity analysis

Funnel plot assessment showed that the studies of Scotté *et al*.^24^ and Kumar *et al*.^18^ were outliers (*Fig. S1*). The meta-analysis was re-rerun with these studies excluded. This showed a reduction in rates of surgery in the included studies (risk difference (RD) −0.11, 95% c.i. −0.17 to −0.06; *Fig. S2*). Assessment following the removal of studies at risk of sequence allocation bias yielded non-significant results (RD −0.07, 95% c.i. −0.15 to 0.01; *Fig. S3*), as did the exclusion of studies with risk of allocation concealment bias (RD −0.06, 95% c.i. −0.17 to 0.04; *Fig. S4*) and incomplete outcome reporting (RD −0.4, 95% c.i. −0.15 to 0.08; *Fig. S5*).

## Discussion

The key finding from the present systematic review was that pooled data do not support the use of WSCA to prevent the need for surgery in patients with aSBO. Despite the common use of WSCA by surgeons in a conservative approach to the management of aSBO^23^, such a strategy did not seem to be superior to standard care (that is, no use of WSCA) in the available randomized clinical trials in the published literature. Furthermore, there were multiple challenges to the external validity of these published trials.

Previous iterations of guidelines have strongly recommended the use of WSCA in aSBO^4^. This review does not support such practice. There are likely multiple reasons for this lack of evidence for such a strategy. First, this review tightly defined the inclusion criteria, meaning that a commonly cited trial that used WSCA in partial SBO was not included^7^, and neither was a trial that instituted WSCA after a prolonged trial of non-operative management of SBO^5^. These trials have both contributed to the favouring of WSCA in aSBO in meta-analyses, but do not reflect the way this intervention is used in modern practice. The literature recommends surgery within 72 h of diagnosis^25^, so using this intervention for people who have already crossed this time point is not generalizable. Second, a large randomized clinical trial has been conducted in the past decade^24^ that is the largest trial to date, almost double in size of the next biggest study. That trial used CT scans without evidence of ischaemia as an entry requirement^24^, which reflects how patients are usually selected for WSCA use at present^26^, and showed that WSCA was not associated with a reduced rate of surgery. The inaccuracy of the diagnostic tool for inclusion may explain the perceived success of WSCA in early trials, where aSBO was not the pathology being treated^27^.

The requirement for surgery was a well-documented outcome in the studies included in the present analysis. Unfortunately, other outcomes of interest, such as small bowel resection, the occurrence of small bowel ischaemia, and mortality, were scarcely reported. These outcomes are important to both surgeons and patients, and are reflected in the recent core outcome set for aSBO^28^. Notably, the triggers for surgical intervention were not standardized across the studies, and allocation concealment and blinding were rare. Along with the typically single-centre nature of the included studies, this raises issues around the impact of individual surgeon behaviours on trial outcomes.

PRECIS-2 assessment provides a useful assessment of included studies. In the present study, PRECIS-2 assessment demonstrated that, for the most part, the included studies were pragmatic studies. There was a tendency towards single-centre studies, limiting the external validity of the findings. It is also recognized that single-centre studies can overestimate effect sizes, leading to excessively positive results^29^. The bias assessment also raises concerns about many of the studies included. The fragility assessment shows that small numbers of patients changing their outcome would alter the overall results of the trial, indicating a sample size too small to detect a robust effect. Although one can accept there are major challenges around the conduct of emergency surgery research, this does not mean that quality and rigour in trials should be sacrificed for ease of delivery. Should there be concerns about the design of future trials in the field, implementation of qualitative work with patients and clinicians may prove illuminating^30^. This is a relatively low evidence area, and patients are in need of improved evidence to reduce the high rates of morbidity and mortality^31^.

Sensitivity analysis showed that only exclusion of trials with perceived risk of publication bias affected outcome, and not bias assessment. The impact of censoring small studies on results means that the evidence for this intervention is not robust. Furthermore, the censoring of the paper by Scotté *et al.*^24^ meant that the study with the most accurate diagnostic modality for SBO was excluded, which raises further concerns about the literature.

As with all systematic reviews, the quality of evidence is limited by the collective quality of the included trials. These have issues around bias in their conduct and limited sample size. The authors acknowledge variation in how WSCA is used in practice. The framing of the research question in the present study reflects the practice in their home nation. Acknowledging this, the review has been conducted according to best practice, with prospective registration, well developed search strategies, and additional assessments from a trial methodology perspective. This adds to insights gleaned from this reassessment of the data.

Based on the findings of the present study, the evidence does not support WSCA as a therapeutic agent for patients with aSBO. It may provide some diagnostic utility in making decisions for surgery at follow-up abdominal radiography^6^. Because the synthesis of data from available clinical trials is not in keeping with the practice of many surgeons^26^, further trials within the modern era (including the use of CT imaging as the standard) are warranted. The present systematic review supports equipoise for such future trials. A large multicentre placebo-controlled trial is required to fully assess the therapeutic efficacy of WSCA in aSBO.

## Supplementary Material

zraf049_Supplementary_Data

## Data Availability

The data underlying this article are available in the article and in its online *Supplementary material*.

## References

[1] ten Broek RPG, Issa Y, van Santbrink EJP, Bouvy ND, Kruitwagen RFPM, Jeekel J et al Burden of adhesions in abdominal and pelvic surgery: systematic review and met-analysis. BMJ 2013;347:f5588 24092941 10.1136/bmj.f5588 PMC3789584

[2] Lee MJ, Sayers AE, Drake TM, Marriott PJ, Anderson ID, Bach SP et al National prospective cohort study of the burden of acute small bowel obstruction. BJS Open 2019;3:354–366 31183452 10.1002/bjs5.50136 PMC6551410

[3] Loftus T, Moore F, VanZant E, Bala T, Brakenridge S, Croft C et al A protocol for the management of adhesive small bowel obstruction. J Trauma Acute Care Surg 2015;78:13–19. discussion 19–21 25539198 10.1097/TA.0000000000000491 PMC5125021

[4] Ten Broek RPG, Krielen P, Di Saverio S, Coccolini F, Biffl WL, Ansaloni L et al Bologna guidelines for diagnosis and management of adhesive small bowel obstruction (ASBO): 2017 update of the evidence-based guidelines from the world society of emergency surgery ASBO working group. World J Emerg Surg 2018;13:24 29946347 10.1186/s13017-018-0185-2 PMC6006983

[5] Choi H-K, Chu K-W, Law W-L. Therapeutic value of Gastrografin in adhesive small bowel obstruction after unsuccessful conservative treatment: a prospective randomized trial. Ann Surg 2002;236:1–6 12131078 10.1097/00000658-200207000-00002 PMC1422541

[6] Ceresoli M, Coccolini F, Catena F, Montori G, Di Saverio S, Sartelli M et al Water-soluble contrast agent in adhesive small bowel obstruction: a systematic review and meta-analysis of diagnostic and therapeutic value. Am J Surg 2016;211:1114–1125 26329902 10.1016/j.amjsurg.2015.06.012

[7] Assalia A, Schein M, Kopelman D, Hirshberg A, Hashmonai M. Therapeutic effect of oral Gastrografin in adhesive, partial small-bowel obstruction: a prospective randomized trial. Surgery 1994;115:433–437 8165534

[8] The health products regulatory authority . https://www.hpra.ie/docs/default-source/Shortages-Docs/2024-11-13-resolved-shortages.pdf?sfvrsn=2 (accessed 27 March 2025)

[9] Higgins JPT, Thomas J, Chandler J, Cumpston M, Li T, Page MJ et al (editors). *Cochrane Handbook for Systematic Reviews of Interventions* version 6.5 (updated August 2024). Cochrane, 2024. https://training.cochrane.org/handbook/current (accessed 11 November 2024)

[10] PRISMA. *PRISMA 2020 Checklist*. https://www.prisma-statement.org/prisma-2020-checklist (accessed 16 November 2024)

[11] Balduzzi S, Rücker G, Schwarzer G. How to perform a meta-analysis with R: a practical tutorial. Evid Based Ment Health 2019;22:153–160 31563865 10.1136/ebmental-2019-300117 PMC10231495

[12] Lin L, Xing A, Chu H, Murad MH, Xu C, Baer BR et al Assessing the robustness of results from clinical trials and meta-analyses with the fragility index. Am J Obstet Gynecol 2023;228:276–282 36084702 10.1016/j.ajog.2022.08.053 PMC9974556

[13] Loudon K, Treweek S, Sullivan F, Donnan P, Thorpe KE, Zwarenstein M. The PRECIS-2 tool: designing trials that are fit for purpose. BMJ 2015;350:h2147 25956159 10.1136/bmj.h2147

[14] Farid M, Fikry A, El Nakeeb A, Fouda E, Elmetwally T, Yousef M et al Clinical impacts of oral Gastrografin follow-through in adhesive small bowel obstruction (SBO). J Surg Res 2010;162:170–176 19524265 10.1016/j.jss.2009.03.092

[15] Haule C, Ongom PA, Kimuli T. Efficacy of Gastrografin^®^ compared with standard conservative treatment in management of adhesive small bowel obstruction at Mulago National Referral hospital. J Clin Trials 2013;3;1000144 24729947 10.4172/2167-0870.1000144 PMC3982137

[16] Biondo S, Parés D, Mora L, Martí Ragué J, Kreisler E, Jaurrieta E. Randomized clinical study of Gastrografin administration in patients with adhesive small bowel obstruction. Br J Surg 2003;90:542–546 12734858 10.1002/bjs.4150

[17] Burge J, Abbas SM, Roadley G, Donald J, Connolly A, Bissett IP et al Randomized controlled trial of Gastrografin in adhesive small bowel obstruction. ANZ J Surg 2005;75:672–674 16076330 10.1111/j.1445-2197.2005.03491.x

[18] Kumar P, Kaman L, Singh G, Singh R. Therapeutic role of oral water soluble iodinated contrast agent in postoperative small bowel obstruction. Singapore Med J 2009;50:360–364 19421678

[19] Fevang BT, Jensen D, Fevang J, Søndenaa K, Ovrebø K, Røkke O et al Upper gastrointestinal contrast study in the management of small bowel obstruction—a prospective randomised study. Eur J Surg 2000;166:39–43 10688215 10.1080/110241500750009681

[20] Lee JF-Y, Meng WC-S, Leung K-L, Yu SC-H, Poon C-M, Lau W-Y.et al Water soluble contrast follow-through in the management of adhesive small bowel obstruction: a prospective randomized trial. Ann Coll Surg Hong Kong 2004;8:120–126

[21] Khorshidi HR, Majidi P, Pirdehghan A. Therapeutic effect of Gastrografin and predictors of operative intervention in patients with adhesive small bowel obstruction: a randomized controlled study. Turk J Surg 2019;35:131–135 32550318 10.5578/turkjsurg.4237 PMC6796074

[22] Rahmani N, Mohammadpour RA, Khoshnood P, Ahmadi A, Assadpour S. Prospective evaluation of oral Gastrografin^®^ in the management of postoperative adhesive small bowel obstruction. Indian J Surg 2013;75:195–199 10.1007/s12262-012-0479-7 PMC3689375 24426426

[23] Di Saverio S, Catena F, Ansaloni L et al Water-soluble contrast medium (Gastrografin) value in adhesive small intestine obstruction (ASIO): a prospective, randomized, controlled, clinical trial. World J Surg 2008;32:2293–2304 18688562 10.1007/s00268-008-9694-6

[24] Scotté M, Mauvais F, Bubenheim M, Cossé C, Suaud L, Savoye-Collet C et al Use of water-soluble contrast medium (Gastrografin) does not decrease the need for operative intervention nor the duration of hospital stay in uncomplicated acute adhesive small bowel obstruction? A multicenter, randomized, clinical trial (adhesive small bowel obstruction study) and systematic review. Surgery. 2017;161:1315–1325 28087066 10.1016/j.surg.2016.11.026

[25] Peacock O, Bassett MG, Kuryba A, Walker K, Davies E, Anderson I et al Thirty-day mortality in patients undergoing laparotomy for small bowel obstruction. Br J Surg 2018;105:1006–1013 29603126 10.1002/bjs.10812

[26] Lee MJ, Sayers AE, Wilson TR, Acheson AG, Anderson ID, Fearnhead NS et al Current management of small bowel obstruction in the UK: results from the national audit of small bowel obstruction clinical practice survey. Colorectal Dis 2018;20:623–630 29331086 10.1111/codi.14016

[27] Maglinte DD, Reyes BL, Harmon BH, Kelvin F M, Turner W W, Hage JE et al Reliability and role of plain film radiography and CT in the diagnosis of small-bowel obstruction. AJR Am J Roentgenol 1996;167:1451–1455 8956576 10.2214/ajr.167.6.8956576

[28] Tripartite Gastrointestinal Recovery SBO Group . A core outcome set for clinical studies of adhesive small bowel obstruction. Colorectal Dis 2022;24:1204–1210 35445534 10.1111/codi.16158 PMC9796004

[29] Bafeta A, Dechartres A, Trinquart L, Yavchitz A, Boutron I, Ravaud P et al Impact of single centre status on estimates of intervention effects in trials with continuous outcomes: meta-epidemiological study. BMJ 2012;344:e813 22334559 10.1136/bmj.e813 PMC3279328

[30] Twiddy M, Birtwistle J, Edmondson A, Croft J, Gordon K, Meads D et al Recruiting to surgical trials in the emergency setting: using a mixed methods study to understand clinician and patient perspectives. BJS Open 2022;6:zrac137 36417312 10.1093/bjsopen/zrac137 PMC9683391

[31] EMSurg Collaborators . Methodological overview of systematic reviews to establish the evidence base for emergency general surgery. Br J Surg 2017;104:513–524 28295254 10.1002/bjs.10476 PMC5363346

