# Supplementary material for: Water-soluble contrast agents in adhesional small bowel obstruction: meta-analysis and PRECIS-2 assessment of trials
Source: BJS Open. 2025 May 9;9(3):zraf049. doi: 10.1093/bjsopen/zraf049 (PMC12062880; doi:10.1093/bjsopen/zraf049)
Supplement: zraf049_Supplementary_Data [file zraf049_supplementary_data.zip › Supplementary_Material.docx]

**Water-Soluble Contrast Agents in Adhesional Small Bowel Obstruction: systematic review, meta-analysis, and PRECIS-2 assessment of trials**

**Authors**

Matthew Gowell^a^, Daniel Baker^b^, Greta McLachlan^c^, David N Naumann^a,e,f^, Adam Peckham-Cooper^b^, Neil Smart^g^, Matthew Lee^a,h^

**Affiliations**

^a^ Department of Trauma and Emergency General Surgery, University Hospitals Birmingham NHS Foundation Trust, Queen Elizabeth Hospital Birmingham, Birmingham, UK

^b^ Leeds Institute for Emegency Surgery, Leeds Teaching Hospitals NHS Trust, Leeds, UK

^c^Department of Surgery, Frimley Health Foundation Trust, Frimley Park Hospital, Portsmouth Rd, Frimley, Camberley, UK

^e^University of Birmingham, Birmingham, UK

^f^Academic Department of Military Surgery and Trauma, Royal Centre for Defence Medicine, Queen Elizabeth Hospital Birmingham, Birmingham, UK

^g^Royal Devon and Exeter NHS Foundation Trust

^h^Department of Applied Health Sciences, College of Medicine and Health | University of Birmingham

**Corresponding author**

Matthew Lee, Department of Applied Health Sciences, College of Medicine and Health | University of Birmingham

Email: [m.j.lee.1@bham.ac.uk](mailto:m.j.lee.1@bham.ac.uk)

**Supplementary Materials – Index**

**Appendix A: Medline Search Strategy page 3**

| **Supplementary Figures and Tables** |  |
| --- | --- |
| Fig S1: Funnel plot page 4 |  |
| Fig S2: Forest plot after exclusion of outliers on funnel plot page 4  Fig S3: Forest plot after exclusion of studies at risk of sequence allocation bias page 5  Fig S4: Forest plot after exclusion of studies at risk of allocation concealment bias page 5  Fig S5: Forest plot after exclusion of studies at risk of incomplete outcome reporting bias page 6  Table S1: Excluded full texts of note page 7 |  |
|  |  |

Appendix A: Medline search strategy

| **Database:** | **Ovid MEDLINE(R) ALL <1946 to July 25, 2024>** | **Results per line:** | **Number of results:** |
| --- | --- | --- | --- |
| **Date:** | **26/07/2024** |  |  |
| 1 | exp Intestinal Obstruction/ | 51322 | **126** |
| 2 | ((bowel* or intestin* or ileu* or mechanical) adj3 (obstruct* or block*)).ti,ab,kw,kf. | 36529 |  |
| 3 | (SBO or ASBO).ti,ab,kw,kf. | 1753 |  |
| 4 | 1 or 2 or 3 | 69792 |  |
| 5 | exp Contrast Media/ | 134480 |  |
| 6 | (soluble adj2 contrast*).ti,ab,kw,kf. | 1951 |  |
| 7 | WSC.ti,ab,kw,kf. | 770 |  |
| 8 | (sodium diatrizoate or meglumine diatrizoate or gastrografin or gastrograffin or urografin or urografphin or amidotrizoate).ti,ab,kw,kf. | 1665 |  |
| 9 | 5 or 6 or 7 or 8 | 136705 |  |
| 10 | 4 and 9 | 1938 |  |
| 11 | Randomized Controlled Trial.pt. | 617552 |  |
| 12 | Controlled Clinical Trial.pt. | 95576 |  |
| 13 | Clinical Trial.pt. | 540238 |  |
| 14 | exp Clinical Trials as Topic/ | 394714 |  |
| 15 | Placebos/ | 35976 |  |
| 16 | Random Allocation/ | 107423 |  |
| 17 | Double-Blind Method/ | 179535 |  |
| 18 | Single-Blind Method/ | 33751 |  |
| 19 | Cross-Over Studies/ | 57102 |  |
| 20 | ((random$ or control$ or clinical$) adj3 (trial$ or stud$)).tw. | 1677139 |  |
| 21 | (random$ adj3 allocat$).tw. | 48577 |  |
| 22 | placebo$.tw. | 258288 |  |
| 23 | ((singl$ or doubl$ or trebl$ or tripl$) adj (blind$ or mask$)).tw. | 206744 |  |
| 24 | (crossover$ or (cross adj over$)).tw. | 104783 |  |
| 25 | 11 or 12 or 13 or 14 or 15 or 16 or 17 or 18 or 19 or 20 or 21 or 22 or 23 or 24 | 2532963 |  |
| 26 | 10 and 25 | **126** |  |

**SF1: Funnel plot of all studies based on primary outcome**

**
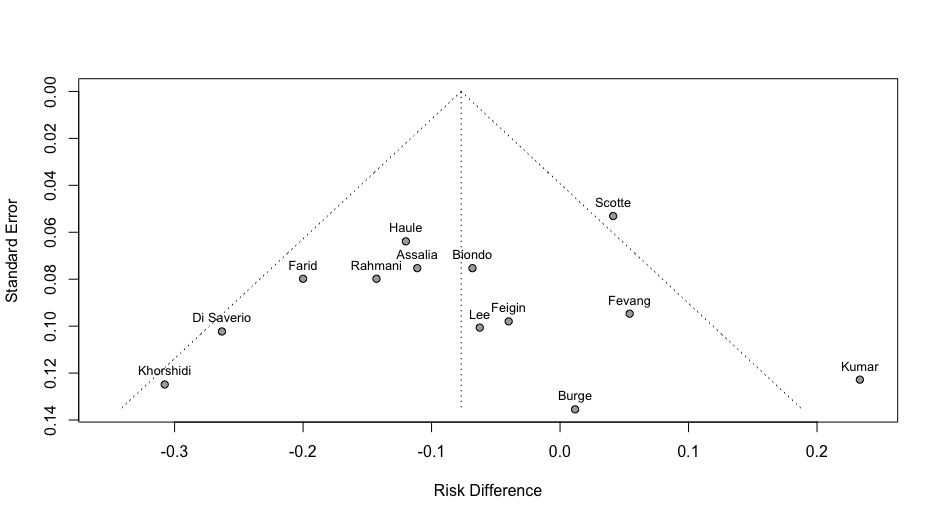
**

SF2: Forest plot after exclusion of outliers from funnel plot

**
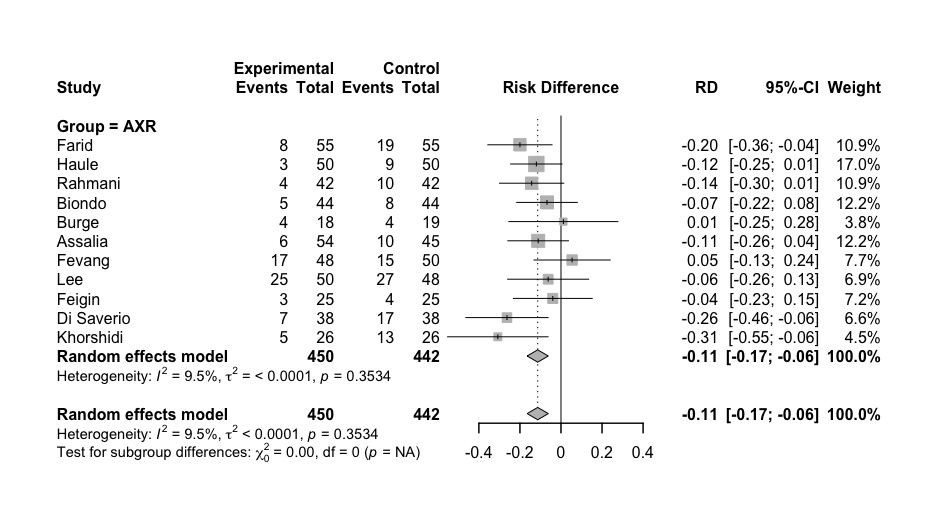
**

**SF3: Forest plot after exclusion of studies at risk of sequence allocation bias**

**
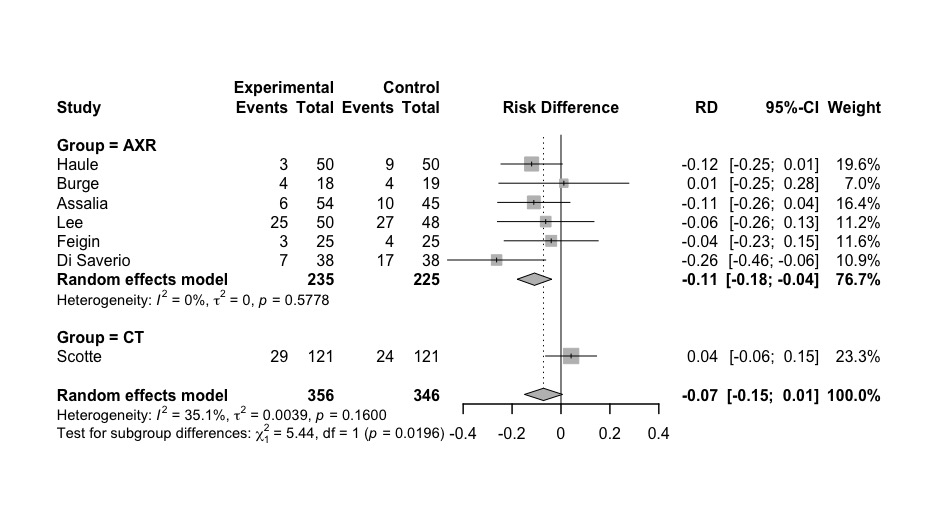
**

**SF4: Forest plot after exclusion of studies at risk of allocation concealment bias**

**
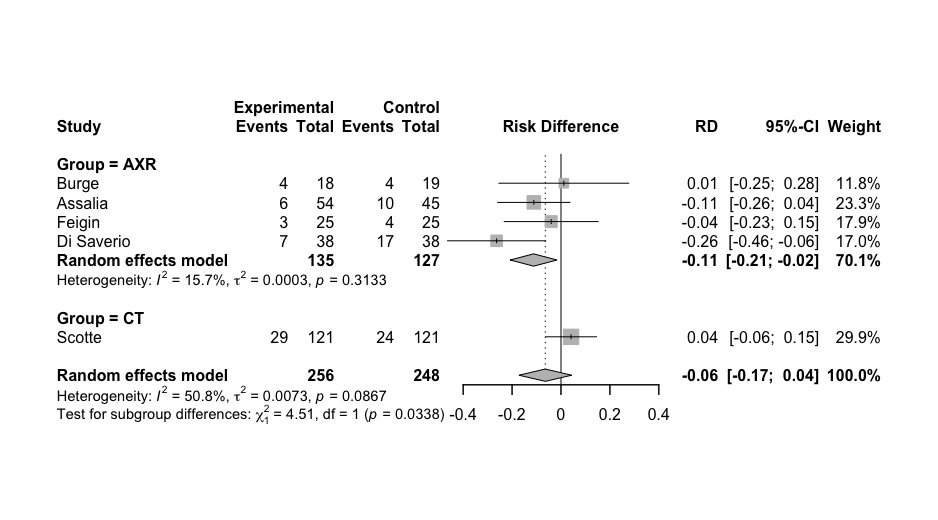
**

**SF5: Forest plot after exclusion of studies at risk of incomplete outcome reporting bias**


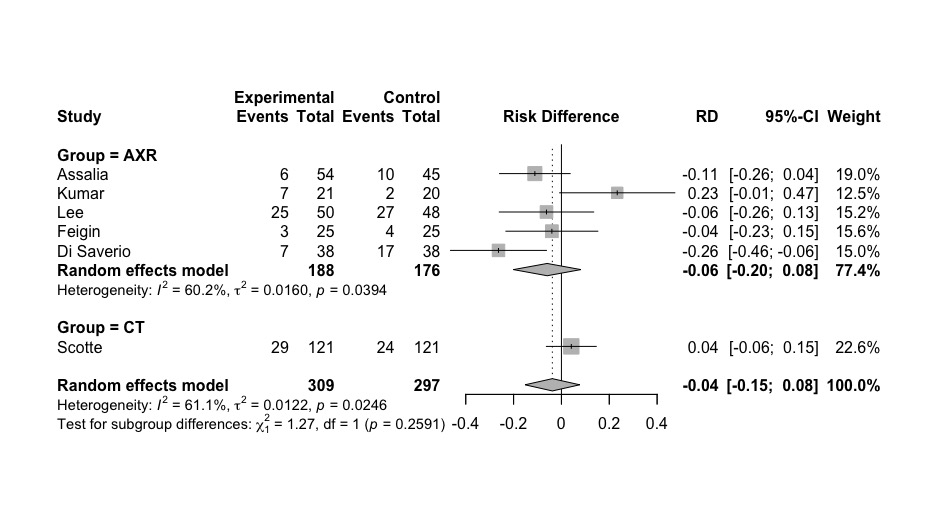


**ST1: Excluded full texts of note**

| **First author** | **Year** | **Title** | **Reason for exclusion** |
| --- | --- | --- | --- |
| Assalia | 1994 | Therapeutic effect of oral Gastrografin in adhesive, partial small-bowel obstruction: A prospective randomized trial | Included partial SBO only |
| Vakil | 2007 | Role of water-soluble contrast study in adhesive small bowel obstruction: a randomized controlled study | Included patients as young as 12; wrong population for this review |
| Brochwicz-Lewinski | 2003 | Small bowel obstruction - The water-soluble follow-through revisited | Unable to retrieve English text |
| Choi | 2002 | Therapeutic Value of Gastrografin in Adhesive Small Bowel Obstruction After Unsuccessful Conservative Treatment A Prospective Randomized Trial | Intervention used after trial of 48+ hours conservative management |
| Katano | 2020 | The first management using intubation of a nasogastric tube with Gastrografin enterography or long tube for non-strangulated acute small bowel obstruction: a multicenter, randomized controlled trial | Long tube not widely used; not appropriate comparator |
